# Distribution of anti‐factor Xa activity in patients with nonvalvular atrial fibrillation receiving 15 mg dose of edoxaban

**DOI:** 10.1002/joa3.13139

**Published:** 2024-08-27

**Authors:** Shotaro Hiramatsu, Hiroyuki Osanai, Yuichiro Sakai, Yoshiki Sogo, Yuki Tanaka, Hikari Matsumoto, Shun Miyamoto, Kensuke Tagahara, Kenji Arai, Takashi Watanabe, Yusuke Sakamoto, Teruhiro Sakaguchi, Shioh Oguchi, Takahiro Kanbara, Yoshihito Nakashima, Hiroshi Asano, Masayoshi Ajioka

**Affiliations:** ^1^ Department of Cardiology Gifu Prefectural Tajimi Hospital Tajimi Gifu Japan; ^2^ Department of Cardiology Tosei General Hospital Seto Aichi Japan; ^3^ Department of Cardiology Kumiai Kosei Hospital Takayama Gifu Japan; ^4^ Department of Cardiology Nagoya University Hospital Nagoya Aichi Japan; ^5^ Department of Cardiology Japanese Red Cross Aichi Medical Center Nagoya Daiichi Hospital Nagoya Aichi Japan

**Keywords:** anticoagulant, anti‐factor Xa, atrial fibrillation, edoxaban, stroke

## Abstract

**Background:**

The distribution of anti‐factor Xa activity (AXA) in patients with nonvalvular atrial fibrillation (NVAF) taking edoxaban 15 mg has not been fully elucidated.

**Methods and Results:**

The trough and peak AXA were measured in 19 NVAF patients taking edoxaban 15 mg. We compared these results with those in patients taking edoxaban 30 mg. The peak AXA differed significantly between the 15 mg and the 30 mg groups (0.74 ± 0.40 IU/mL vs. 1.25 ± 0.48 IU/mL, respectively; *p* < 0.0001).

**Conclusions:**

Peak but trough AXA in the patients receiving edoxaban 15 mg were significantly lower than those in patients receiving edoxaban 30 mg.

## Graphical abstract

The trough and peak AXA were measured in 19 NVAF patients taking edoxaban 15 mg. We compared these results with those in patients taking edoxaban 30 mg. Peak but trough AXA in the patients receiving edoxaban 15 mg were significantly lower than those in patients receiving edoxaban 30 mg.

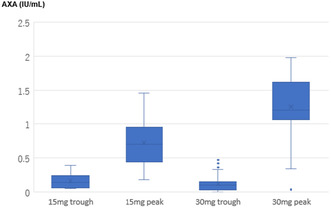

## 1 INTRODUCTION

Edoxaban is a selective factor‐Xa (FXa) inhibitor for the prevention of stroke in patients with nonvalvular atrial fibrillation (NVAF).^1^, ^2^, ^3^ Since 2021, edoxaban 15 mg dose has been approved in Japan as a new dose for very elderly patients with high bleeding risks. It is recommended for patients aged 80 years or older and had to be considered ineligible for any oral anticoagulants at the approved dose for one or more of the following reasons: low creatinine clearance (15–30 mL per minute), history of bleeding from a critical area/organ or gastrointestinal bleeding, low body weight (≤45 kg), continuous use of nonsteroidal anti‐inflammatory drugs (NSAIDs), and current use of an antiplatelet drug. In very elderly Japanese patients with NVAF, 15 mg dose of edoxaban was superior to placebo in preventing stroke or systemic embolism and did not result in a significantly higher incidence of major bleeding than placebo.^4^ However, the actual edoxaban concentration has not been elucidated in daily clinical practice. Previous studies have shown that chromogenic anti‐factor Xa activity (AXA) is the most appropriate assay to measure the pharmacodynamics of an FXa inhibitor and to estimate plasma drug concentrations.^5^, ^6^, ^7^, ^8^ The aims of the present study were to determine the distribution of the steady‐state trough and peak AXA values in very elderly Japanese NVAF patients receiving edoxaban 15 mg dose.

## 2 METHODS

### 2.1 Study design and subjects

This observational study of Japanese patients with NVAF on edoxaban‐15 mg therapy was conducted during routine clinical practice at Tosei General Hospital, Aichi, Japan. From March 1, 2022 to September 30, 2022, we obtained consent from 19 patients taking edoxaban 15 mg to participate in this study and recorded their clinical characteristics and the AXA levels. The prothrombin time (PT) values are also evaluated in the part of the patients (in the 15 mg group, 16 of 19 patients, and in the 30 mg group, 27 of 49 patients) by the discretion of the attending physician.

All patients were aged 80 years or older and had one or more of the following facts: low creatinine clearance (15–30 mL per minute), history of bleeding from a critical area/organ or gastrointestinal bleeding, low body weight (≤45 kg), continuous use of NSAIDs, and current use of an antiplatelet drug. Patients provided written informed consent to participate, and this study was conducted in accordance with the ethical policies of Tosei General Hospital.

We also compared these data with AXA levels in patients taking edoxaban 30 mg dose obtained from a previous study we conducted.^9^

Creatinine clearance was determined by the Cockcroft‐Gault formula.^10^

### 2.2 Measuring AXA and PT

The HemosIL Liquid Heparin Kit (Instrumentation Laboratory, Lexington, KY, USA) was used for measuring AXA as previously reported.^7^, ^9^, ^11^ AXA values at trough and peak times after repeated edoxaban intake were measured >72 h after the start of treatment. The trough time was defined as that immediately before the intake of edoxaban, and the peak time was defined as 2 h after the intake of edoxaban. Those time was defined according to a previous report from our institution.^9^ The peak and trough time of PT was also defined as the same time. PT was determined with HemosIL RecombiPlasTin (Instrumentation Laboratory).

### 2.3 Statistical analysis

Categorical variables are presented as numbers and percentages, and continuous variables are presented as mean ± SD. To compare parameters between groups, unpaired *t*‐test and chi‐squared test was used. To compare AXA values and PT values, the Mann–Whitney *U*‐test was used. *p* < 0.05 was considered to indicate statistical significance. All statistical software program (Ekuseru‐Tokei, BellCurve, Tokyo, Japan).

## 3 RESULTS

The characteristics of the patients are shown in Table 1. Nineteen patients receiving edoxaban 15 mg, and 49 patients receiving 30 mg, consistent with the dose recommendation, were included. In the 15 mg group, the mean age was significantly higher (87.5 ± 4.1 vs. 78.5 ± 6.9 years, respectively; *p* < 0.001) and the mean creatinine clearance was significantly lower (28.6 ± 9.4 vs. 44.7 ± 16.7 years, respectively; *p* < 0.001).

**TABLE 1:** Baseline patient characteristics.

	15 mg dose (*n* = 19)	30 mg dose (*n* = 49)	*p* value
Age (years)	87.5 ± 4.1	78.5 ± 6.9	<0.001
Male gender, *n* (%)	11 (57.9)	24 (49.0)	
Body weight (kg)	49.4 ± 8.2	52.0 ± 8.9	0.31
Paroxysmal atrial fibrillation, *n* (%)	8 (36.8)	22 (44.9)	
Serum creatinine (mg/dL)	1.28 ± 0.39	1.05 ± 0.48	0.019
Mean creatinine clearance (mL/min)	28.6 ± 9.4	44.7 ± 16.7	<0.001
Mean CHADS2 score, *n* (%)	3.6 ± 1.0	2.2 ± 1.1	<0.001
0	0	2 (4.1)	
1	1 (5.3)	10 (20.4)	
2	0	21 (42.9)	
≧3	18 (94.7)	16 (32.7)	
Congestive heart failure, *n* (%)	19 (100)	23 (46.9)	<0.001
Hypertension, *n* (%)	16 (84.2)	29 (59.2)	0.065
Age ≧75 years, *n* (%)	19 (100)	36 (73.5)	0.013
Diabetes mellitus, *n* (%)	10 (52.6)	10 (20.4)	0.009
Baseline stroke/transient ischemic attack/systemic embolism, *n* (%)	0	5 (10.2)	0.15
Previous anticoagulants, *n* (%)			
None	4 (21.1)	24 (50.0)	
Warfarin	1 (5.3)	20 (40.8)	
Dabigatran	1 (5.3)	2 (4.1)	
Rivaroxaban	3 (15.8)	2 (4.1)	
Apixaban	1 (5.3)	1 (2.0)	
Edoxaban (30 mg OD)	9 (47.4)	—	—
Peak AXA (IU/mL)	0.74 ± 0.40	1.25 ± 0.48	<0.001
Trough AXA (IU/mL)	0.19 ± 0.12	0.12 ± 0.11	0.080

	15 mg dose (*n* = 16)	30 mg dose (*n* = 27)	*p* value
Peak PT (s)	14.1 ± 1.8	16.2 ± 3.9	0.06
Trough PT (s)	11.9 ± 0.7	11.9 ± 1.6	0.93

*Note*: The PT values were evaluated in the part of the patients (in the 15 mg group, 16 of 19 patients, and in the 30 mg group, 27 of 49 patients) by the discretion of the attending physician.

**FIGURE 1:**
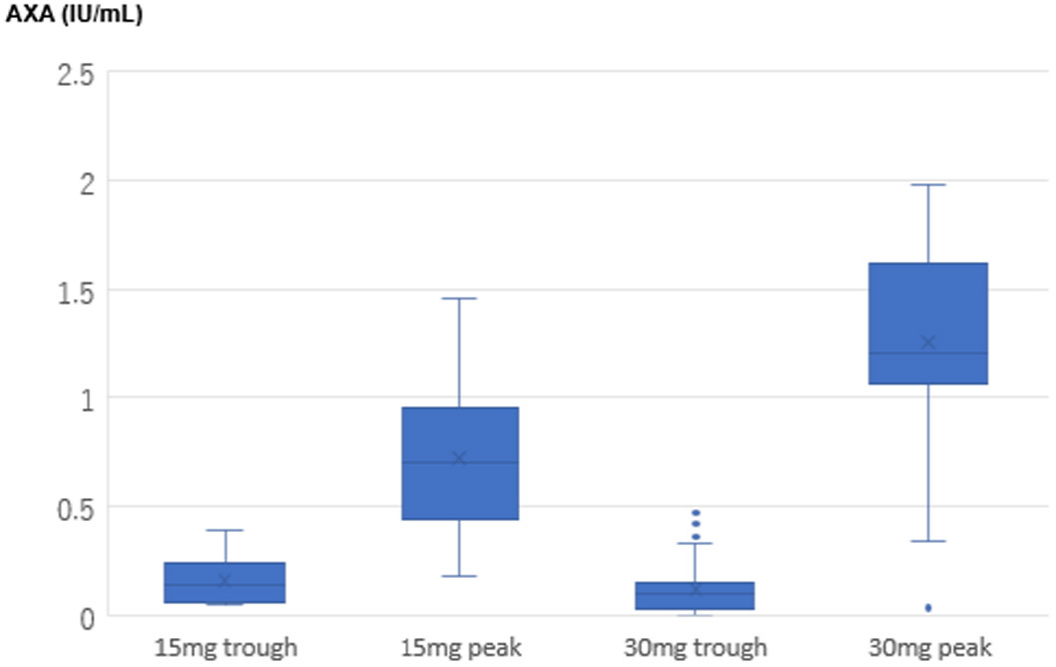
Distribution of steady‐state trough and peak anti‐factor Xa activity (AXA) values of the 15 mg group and the 30 mg group. The trough AXA value did not significantly differ between the groups. On the other hand, the peak AXA in the 15 mg group was significantly lower than that of the 30 mg group. Horizontal line in boxes represents medians. Tops and bottoms of boxes indicate 75th and 25th percentile, respectively. Tops and bottoms of bars indicate maximum and minimum non‐outliers, respectively. Circles indicate outliers above or below 1.5 times interquartile range from the 75th and 25th percentiles.

The reasons for oral anticoagulant ineligibility in the 15 mg group are shown in Table 2. Steady‐state AXA values were compared between edoxaban 15 mg group and 30 mg group and the distribution of the AXA values were shown in Figure 1. The trough AXA values were not significantly different between the two groups (0.19 ± 0.12 vs. 0.12 ± 0.11 IU/mL, respectively; *p* = 0.08). On the other hand, the peak AXA values in the 15 mg group were significantly lower than those of the 30 mg group (0.74 ± 0.40 vs. 1.25 ± 0.48 IU/mL, respectively; *p* < 0.001).

**TABLE 2:** Reasons for oral coagulant ineligibility in the patients taking edoxaban 15 mg OD.

Severe kidney impairment (creatinine clearance <30 mL/min), *n* (%)	4 (21.1)
History of bleeding from critical area or organ, *n* (%)	4 (21.1)
Intracranial	0
Gastrointestinal	0
Intraocular	0
Others^a^	4 (21.1)
Low body weight (≦45 kg), *n* (%)	5 (26.3)
Continuous use of NSAIDs, *n* (%)	0
Use of an antiplatelet drug, *n* (%)	5 (26.3)
Aspirin	0
Clopidogrel	4 (21.1)
Other	1 (5.3)

^a^ The details are as follows (bloody sputum: 2, iron deficiency anemia: 2). The exact source of bleeding is unknown in either patient.

**FIGURE 2:**
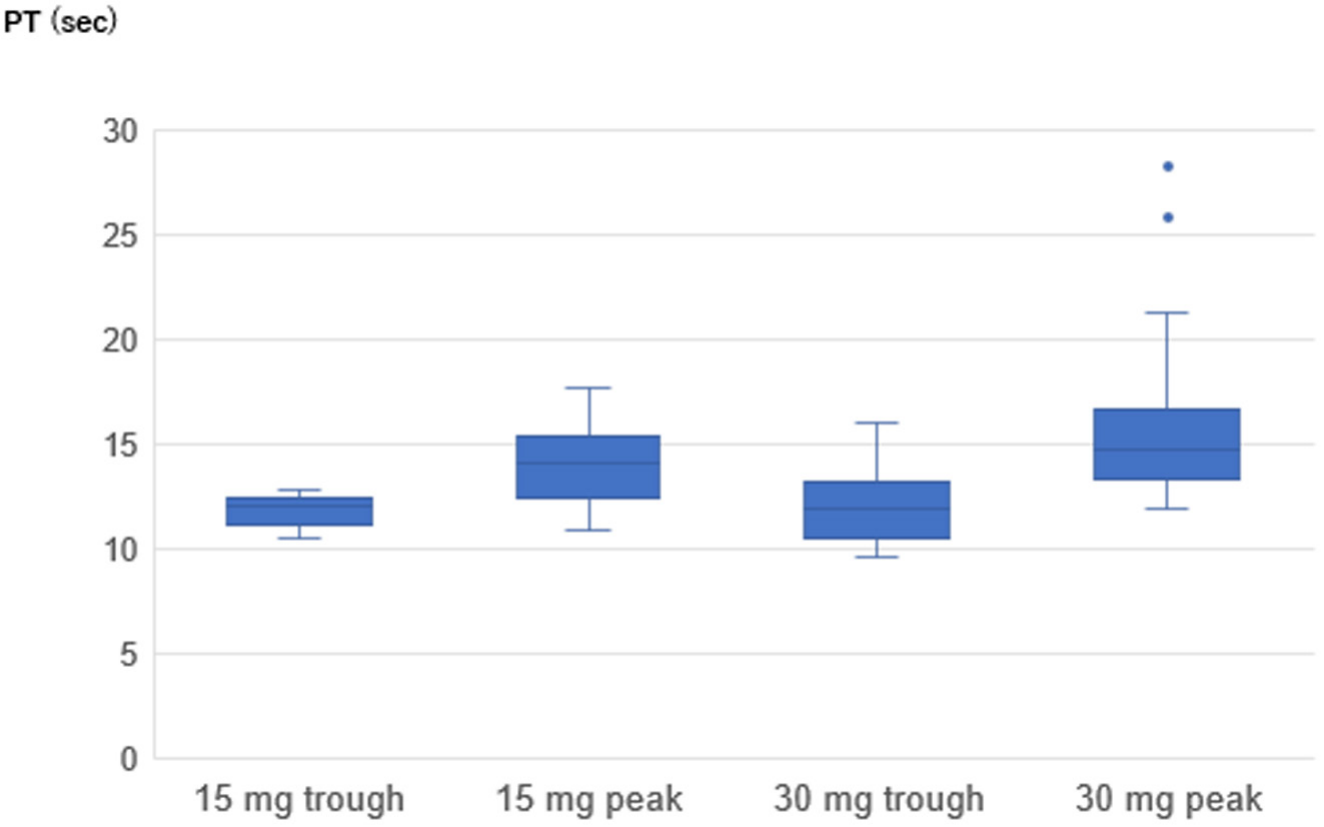
Distribution of trough and peak PT values of the 15 mg group and the 30 mg group. The PT values showed no significant difference between the two groups for both trough and peak values. However, there was a trend of lower peak values observed in the 15 mg group. Horizontal line in boxes represents medians. Tops and bottoms of boxes indicate 75th and 25th percentile, respectively. Tops and bottoms of bars indicate maximum and minimum non‐outliers, respectively. Circles indicate outliers above or below 1.5 times interquartile range from the 75th and 25th percentiles. The PT values were evaluated in the part of the patients (in the 15 mg group, 16 of 19 patients, and in the 30 mg group, 27 of 49 patients) by the discretion of the attending physician.

The PT values were compared between edoxaban 15 mg group and 30 mg group and the distribution of the AXA values were shown in Figure 2. The PT values showed no significant difference between the two groups for both trough and peak values. However, there was a trend of lower peak values observed in the 15 mg group.

## 4 DISCUSSION

In this study, we reported the results of trough and peak AXA values in very elderly Japanese NVAF patients treated with edoxaban 15 mg. To our knowledge, this is the first report of the distribution of steady‐state AXA values in Japanese very elderly patients with NVAF on edoxaban 15 mg dose therapy in daily clinical practice. The main finding of the present study was that the peak AXA levels in edoxaban 15 mg group were significantly lower than those in edoxaban 30 mg. Considering that creatinine clearance of the 15 mg group was significantly lower than that of the 30 mg group, this significant low peak edoxaban concentration in the 15 mg group indicates that 15 mg edoxaban is an extremely low‐dose setting even for very elderly patients who are concerned that drug concentration tends to rise.

Oral anticoagulant treatment for the prevention of stroke in very elderly patients with NVAF is challenging because of comorbid bleeding risks.^12^ In such a situation, the ELDERCARE‐AF trial showed that edoxaban 15 mg dose was superior to placebo in preventing stroke or systemic embolism without significantly increasing major bleeding events for very elderly Japanese patients with NVAF who were not appropriate candidates for standard dose of any oral anticoagulants.^4^ In response to the result of this trial, edoxaban 15 mg dose has been approved in Japan as a new dose for very elderly patients with bleeding risk factors.

The results of our study that the peak AXA levels in edoxaban 15 mg group were significantly lower than those in edoxaban 30 mg were comparable to the results from the pharmacokinetics of edoxaban‐15 mg dose in the ELDERCARE‐AF study.^13^ Edoxaban plasma concentrations in the ELDERCARE‐AF patients were reported slightly higher than the edoxaban‐15 mg group in ENGAGE AF‐TIMI 48, lower than the ENGAGE AF‐TIMI 48 high dose reduced to 30 mg group, but similar to the Japanese severe renal impairment patients.^13^, ^14^, ^15^ Under these pharmacokinetics, edoxaban 15 mg dose has resulted in a significant reduction in stroke events and although not statistically significant, approximately twice as many major bleeding events compared to placebo, suggesting that this dose setting may manage to balance the risk–benefit in very elderly AF patients with high bleeding risk. Furthermore, in the ELDERCARE‐AF study, edoxaban concentration was significantly higher both in the trough and peak in patients with severe renal failure or low body weight, then we should pay attention depending on the patient's background when administering edoxaban 15 mg dose in very elderly patients with high bleeding risk.

In fact, in our study, the trough AXA values in the edoxaban 15 mg dose patients were the same as the trough AXA values in the edoxaban 30 mg dose patients. The result was slightly different from the ELDERCARE‐AF trial, in which the trough edoxaban concentrations were lower than the ENGAGE AF‐TIMI 48 high‐dose reduced to 30 mg group.^4^, ^14^ This difference may be related to the fact that the mean creatinine clearance in our study was lower than the mean creatinine clearance in the ELDERCARE‐AF trial (28.6 ± 9.4 and 36.7 ± 14.3 mL/min, respectively).

The PT values showed no significant difference between the two groups for both trough and peak values. Compared to AXA values, it showed lower sensitivity, but there was a trend of lower peak values. Suzuki et al. evaluated the responses of PT to plasma edoxaban concentration in Japanese NVAF patients taking edoxaban 30 or 60 mg, and reported the correlation coefficient was 0.911 with the same reagent as ours.^16^ In addition, their results showed that the mean trough PT was 12.3 s and the mean peak PT was 16.0 s, which was close to the PT values in the edoxaban 30 mg group in our study especially in the peak.

This study had some limitations. First, it was conducted at a single center with a small number of enrolled patients. Additionally, because of the short observation period in our study and the absence of stroke, systemic embolism or bleeding events, we have not been able to discuss patient outcomes. Furthermore, since the baseline patient characteristics are different between edoxaban 15 mg and 30 mg groups, several factors affecting AXA may be related to other than dose. Second, the peak time was defined as 2 h after drug administration according to a previous report, but the actual peak concentration may differ among individual patients, especially in elderly people with impaired kidney function. Third, the different chromogenic AXA assays available on the market may show different relationships with plasma edoxaban concentration depending on the reagent,^7^ so the results of this study might be different had other reagents been used.

## 5 CONCLUSION

The distribution of AXA values among very elderly Japanese patients with NVAF and who were taking edoxaban 15 mg dose in daily clinical practice was investigated at steady state. The peak AXA values of the 15 mg group were significantly lower than that of the 30 mg group. On the other hand, the trough AXA values were not significantly different between the two groups.

## CONFLICT OF INTEREST STATEMENT

The authors declare that there are no conflicts of interest.

## ETHICS STATEMENT

It conforms to the Declaration of Helsinki and the Ethical Guidelines for Clinical Studies issued by the Ministry of Health, Labour and Welfare, Japan. The present study was approved by the Medical Ethics Committee of Tosei General Hospital. Reference number: no. 1007.

## PATIENT CONSENT STATEMENT

Patients provided written informed consent to participate, and this study was conducted in accordance with the ethical policies of Tosei General Hospital.

## IRB INFORMATION

The present study was approved by the medical ethics committee of Tosei General Hospital. Reference number: No.1007.

## Data Availability

The deidentified participant data will not be shared.
